# Computationally Efficient Cooperative Dynamic Range-Only SLAM Based on Sum of Gaussian Filter †

**DOI:** 10.3390/s20113306

**Published:** 2020-06-10

**Authors:** Jung-Hee Kim, Doik Kim

**Affiliations:** 1 Department of Electronic Engineering, Hanyang University, Seoul 04763, Korea; jhkim.sp.0901@gmail.com; 2 Center for Intelligent and Interactive Robotics, Korea Institute of Science and Technology, Seoul 02792, Korea

**Keywords:** simultaneous localization and mapping (SLAM), range-only SLAM, sum of Gaussian (SoG) filter, cooperative approach

## Abstract

A cooperative dynamic range-only simultaneous localization and mapping (CDRO-SLAM) algorithm based on the sum of Gaussian (SoG) filter was recently introduced. The main characteristics of the CDRO-SLAM are (i) the integration of inter-node ranges as well as usual direct robot-node ranges to improve the convergence rate and localization accuracy and (ii) the tracking of any moving nodes under dynamic environments by resetting and updating the SoG variables. In this paper, an efficient implementation of the CDRO-SLAM (eCDRO-SLAM) is proposed to mitigate the high computational burden of the CDRO-SLAM due to the inter-node measurements. Furthermore, a thorough computational analysis is presented, which reveals that the computational efficiency of the eCDRO-SLAM is significantly improved over the CDRO-SLAM. The performance of the proposed eCDRO-SLAM is compared with those of several conventional RO-SLAM algorithms and the results show that the proposed efficient algorithm has a faster convergence rate and a similar map estimation error regardless of the map size. Accordingly, the proposed eCDRO-SLAM can be utilized in various RO-SLAM applications.

## 1. Introduction

Simultaneous localization and mapping (SLAM), that is, localizing a robot while building a map of unknown environments at the same time, has been a popular topic. It has been developed in several forms, e.g., visual SLAM [1,2], bearing-only SLAM [3], range-only SLAM (RO-SLAM) [4,5,6,7,8,9], and their combinations [10,11], etc.

**Figure 1:**
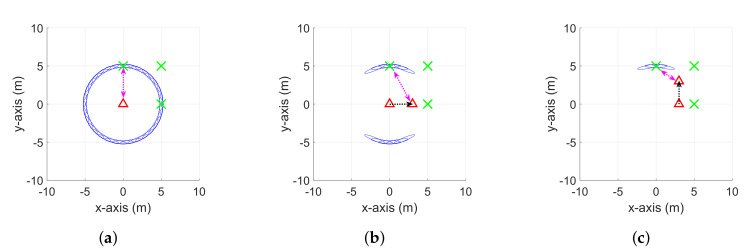
Explanation of the conventional RO-SLAM algorithms. Red triangles and green crosses indicate robots and neighbor nodes, respectively. Black and magenta dotted lines are the movement of the robot and range measurements, respectively. Blue ellipse is a possible location of nodes. The conventional RO-SLAM algorithms involve (**a**) the initial localization when receiving the first measurement, (**b**) movement of a robot to disambiguate the x-axis, and (**c**) movement to disambiguate the y-axis.

Compared with other types of SLAMs, the RO-SLAM, which uses range sensors only, has some distinguishing features. First, the range measurements are highly ambiguous. The potential location of a node can be anywhere on a ring shape, as shown in Figure 1a. Such ambiguity progressively disappears with the additional range measurements at different locations of a robot, as illustrated in Figure 1b,c. Second, the map itself simply shows the locations of nodes. Therefore, the data association issue, which is one of the main difficulties in other types of SLAMs, does not need to be considered. Due to this simplicity, the RO-SLAM has been applied in a wide range of applications such as submarine autonomous vehicles, search and rescue, etc., [6,12].

The Extended Kalman filter (EKF) has been widely employed as a standard solution to the RO-SLAM problem, along with some beacon initialization methods, e.g., trilateration [4], the probability grid [5], the particle filter [7], and the sum of Gaussian (SoG) filter [5,6]. The trilateration method (or multilateration) is simple, but its performance can be seriously damaged by measurement noise. The probability grid can perform better than the trilateration, but its performance depends on the size and resolution of the grid. The particle filter can represent an arbitrary probability density function (pdf) with a number of particles, such as a non-Gaussian ring shape for the beacon location in the RO-SLAM; however, it has a high computational burden due to the large number of particles. Compared with the particle filter approach, the SoG filter [5,6] can give a similar estimation accuracy in a more computationally efficient way, because it can efficiently cover almost the same area of beacon locations with a small number of Gaussian distributions. In this paper, the multilateration and SoG approaches are utilized to determine the initial position to give a fast and accurate localization result.

To improve the estimation accuracy, several RO-SLAM algorithms employ a cooperative approach, in which there are not only direct measurements between a robot and its neighboring nodes, but also, other inter-node measurements are integrated for localization [8,9]. In Ref. [8], the inter-node measurements were simply incorporated into the standard EKF based framework, and the efficiency and scalability of the large scale sensor network were further improved by using the sparse extended information filter (SEIF) based cooperative algorithm [9,13]. Recently, the SoG filter based cooperative approach, called the cooperative dynamic RO-SLAM (CDRO-SLAM) [14], was introduced by using the advantages of the SoG filter, such as the high estimation accuracy, as mentioned above. In addition, the CDRO-SLAM can cope with moving nodes by appropriately manipulating the SoG filter, unlike other RO-SLAM algorithms which assume that nodes are fixed.

In this paper, the efficient implementation of the CDRO-SLAM (eCDRO-SLAM) is proposed by introducing several efficient implementation techniques to mitigate the high computational burden of the CDRO-SLAM due to the inter-node measurements. Furthermore, its detailed computational complexity analysis is also given, and the results show that the computational efficiency of the eCDRO-SLAM is significantly improved over the CDRO-SLAM, which is shown in the experiment results.

The main properties of the proposed eCDRO-SLAM can be summarized, where the first two properties are obtained by inheriting from the advantages of the CDRO-SLAM:

- Accurate and fast localization results are achieved by incorporating the cooperative approach and the SoG filter.
- Sensor nodes can be applied to dynamic environments where installed nodes are not fixed or moving such as unstable and unstructured disaster sites, moving landmarks, interactive information with human and non-fixed objects to analyze human activity, etc.
- To mitigate the high computational cost induced by the inter-node measurements, several efficient techniques are introduced, which greatly reduces the required computational complexity of the proposed eCDRO-SLAM.

This paper is organized as follows: Section 2 derives the CDRO-SLAM, including a cooperative scheme using inter-node measurements and a tracking scheme of non-fixed nodes. In Section 3, an efficient implementation of the CDRO-SLAM (eCDRO-SLAM) is presented to mitigate the computational burden due to the cooperative scheme. Section 4 verifies the performance of the eCDRO-SLAM with several experimental results, and Section 5 gives the concluding remarks.

## 2. Cooperative Dynamic RO-SLAM

### 2.1. Overview

This section summarizes our previous work on the CDRO-SLAM [14], aimed at developing a cooperative RO-SLAM under dynamic environments. The CDRO-SLAM algorithm is composed of two stages: (i) the initialization stage estimates the initial locations of a robot and map by using inter-node measurements, which improves and accelerates the map estimation and (ii) the movement stage further refines the map generated in the initialization stage or tracks nodes in motion.

In more detail, the initialization stage is described in Algorithm 1. (i) Once the initial positions of nodes are guessed with the help of iterative multilateration, it is refined by employing the sum of Gaussian (SoG) filter approach. (ii) The SoG variables are first generated to find the candidate locations of neighbors, and (iii) the SoG variables are updated by exploiting the inter-node measurements to quickly reduce the number of candidates. (iv) The weighted SoG variables are merged into a single Gaussian distribution to estimate the new positions of the nodes. (v) Furthermore, a method to transfer the weight of the SoG variables is applied to resolve the inherent non-convexity in the anchor-free localization. (vi) The above procedures are repeated until the positions of all nodes converge.

**Figure 2:**
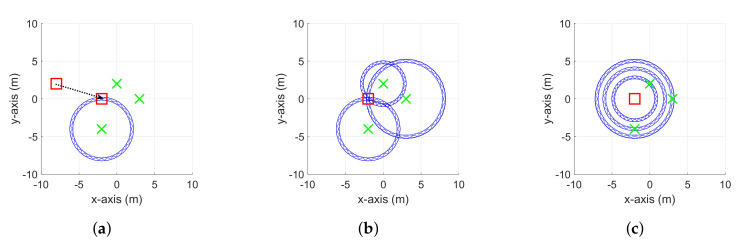
Description of the updated procedures for tracking moving nodes. (**a**) Reset the SoG variables from the neighbor node to the moving node, (**b**) update and merge the SoG variables, and (**c**) reset the SoG variables from the moving node to its neighbor nodes and update the corresponding weights. The red squares and green crosses represent the moving nodes and neighbor nodes, respectively. The black dotted line represents the movement of a node.

In the movement stage, a robot and/or some nodes can be moved. Firstly, if a robot moves, its motion is updated using raw odometry data. Then, after receiving a new distance from the neighbor node, the locations of the corresponding neighbor node and the robot are further refined by using the EKF procedure, like in the usual RO-SLAM algorithms (see [15] for details). Secondly, for the case where some nodes move, their movement can be tracked by properly manipulating the SoG filter, which is illustrated in Algorithm 2 and Figure 2. (i) If *j* denotes an index of a moving node, the movement of the *j*th node is first detected by one of motion detection techniques (e.g., the norm of the accelerometer). (ii) When getting a newly modified distance due to the motion of the *j*th node, the corresponding SoG variables are reset (see Figure 2a). After that, the new position of the *j*th node is estimated by (iii) updating and (iv) merging the SoG variables (see Figure 2b). Finally, the SoG variables from the *j*th node to its neighbor nodes are also recalculated by (v) resetting and (vi) updating them to reflect the newly estimated position of the *j*th node (see Figure 2c).

**Figure 3:**
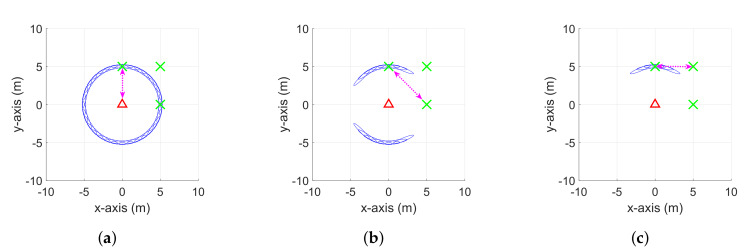
Explanation of the weight update scheme in the CDRO-SLAM. Red triangles and green crosses indicate robots and neighbor nodes, respectively. Black and magenta dotted lines are the movement of the robot and range measurements, respectively. Blue ellipse is a possible location of nodes. The CDRO-SLAM involves (**a**) the initialization of the SoG variables when receiving the first measurement, (**b**) determining the SoG distribution after updating the weight with the first inter-node measurement, and (**c**) the second inter-node measurement.

Differences between the conventional SoG based RO-SLAM algorithms and the proposed CDRO-SLAM are discussed in terms of the weight update scheme and computational complexity. Figure 1 and Figure 3 show the weight update processes of both approaches, respectively. For both algorithms, the weight update starts when the first measurement between a robot and one of its neighbor nodes is received, and then the potential location is assumed to be anywhere within a ring shape whose radius is equal to the measurement (see Figure 1a and Figure 3a). For conventional algorithms, the ambiguity progressively disappears by moving a robot along with x and y axes, i.e., a non-collinear motion (see Figure 1b,c). On the other hand, the proposed algorithm updates the weight by using inter-node measurements without moving a robot. The ambiguity is gradually removed as more measurements from the non-collinear adjacent nodes arrive (see Figure 3b,c).

The computational cost of the CDRO-SLAM is increased due to the inclusion of the SoG variables for inter-node measurements. To reduce this computational burden, several efficient implementation techniques such as the weight symmetry of the SoG distributions are introduced later in Section 3.
**Algorithm 1:** Initialization stage of the CDRO-SLAM.
(*M* for the number of nodes, and i,j,m for the indices of nodes)
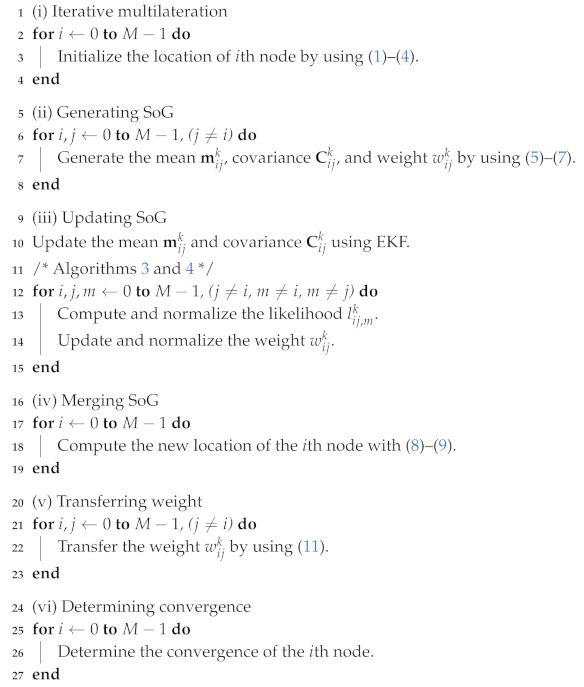

**Algorithm 2:** Moving stage of the cooperative dynamic range-only simultaneous localization and mapping (CDRO-SLAM).
(*M* represents the number of nodes, and i,j,m represent the indices of nodes)
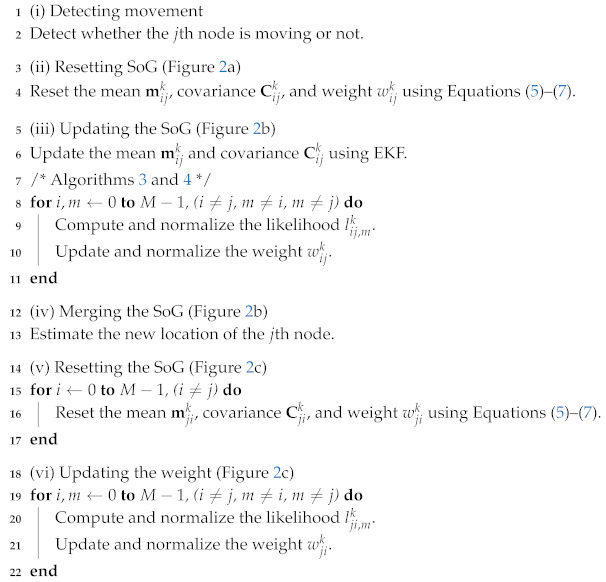


### 2.2. Initialization Stage

#### 2.2.1. Iterative Multilateration

The initial estimation is done by employing the modified iterative multilateration method so that errors are not propagated in each iteration. As stated in [16], the first three nodes are supposed to be placed at the origin, on the positive x-axis, and in the upper half plane, respectively, as follows:

(1) $$\begin{array}{cc} p_{0}\left(n\right) & =(x_{0}\left(n\right),y_{0}\left(n\right))=\left(0,0\right) \end{array}$$

(2) $$\begin{array}{cc} p_{1}\left(n\right) & =\left(x_{1}\left(n\right),y_{1}\left(n\right)\right)=\left(d_{01},0\right) \end{array}$$

(3) $$\begin{array}{cc} p_{2}\left(n\right) & =(x_{2}\left(n\right),y_{2}\left(n\right))\\ & =\left(\frac{d_{01}^{2}+d_{02}^{2}-d_{12}^{2}}{2x_{1}\left(n\right)},\sqrt{d_{02}^{2}-x_{2}^{2}\left(n\right)}\right). \end{array}$$

In Equations (1)–(), $p_{i}\left(n\right)$ is defined as the position of the *i*th node at iteration *n*, and $d_{ij}$ is the distance measurement between the *i*th and *j*th nodes. The next node is localized by exploiting the positions of the first two nodes as in $p_{2}\left(n\right)$, resulting in the following two candidates due to its ambiguity:

(4) $$\begin{array}{cc} p_{m}\left(n\right) & =(x_{m}\left(n\right),y_{m}\left(n\right))\\ \; & =\left(\frac{d_{01}^{2}+d_{0m}^{2}-d_{1m}^{2}}{2x_{1}\left(n\right)},\pm \sqrt{d_{0m}^{2}-x_{m}^{2}\left(n\right)}\right). \end{array}$$

The sign of $y_{m}\left(n\right)$ can be chosen as the one whose estimated distance from $p_{m}\left(n\right)$ to $p_{2}\left(n\right)$ is closer to the measurement $d_{2m}$. Note that $p_{m}\left(n\right)$ is affected by the position error of $p_{1}\left(n\right)$, as shown in Equation (4). The above procedures (i.e., Equations (1)–(4)) are used in Algorithm 1.

#### 2.2.2. Generating SoG

**Figure 4:**
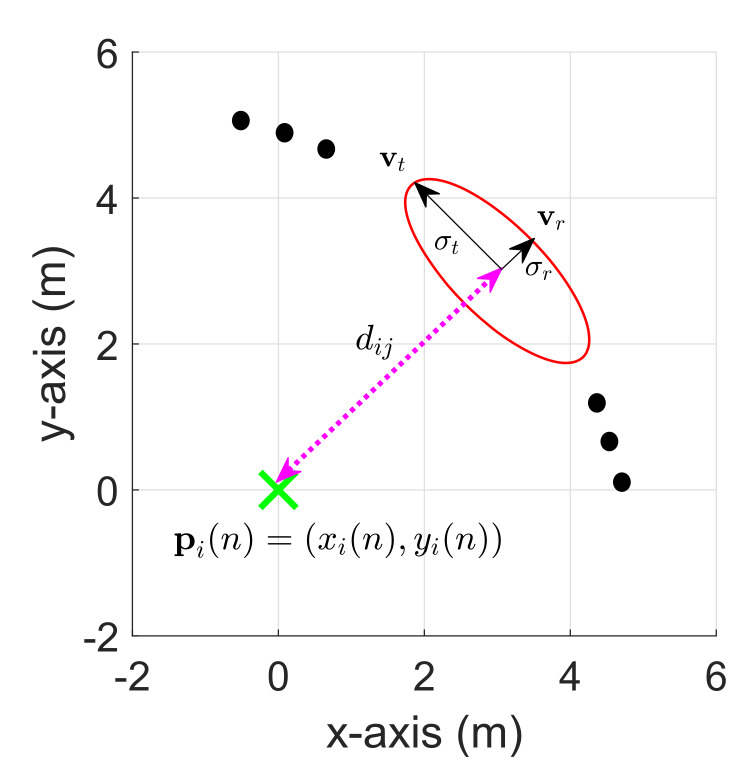
Generation of SoG variables. The dotted magentaline represents the measurement. $v_{r}$ and $v_{t}$ are, respectively, the radial and tangential unit vectors, and $\sigma_{r}$ and $\sigma_{t}$ denote, respectively, the radial and tangential standard deviations.

After guessing the initial positions of a robot and map, SoG variables are generated as shown in Figure 4. Note that in the conventional SoG based RO-SLAM methods, only the SoG variables between robot and its neighbor nodes are generated, while in the proposed CDRO-SLAM, those between any pair of two nodes are generated. For two different nodes (let us say the *i*th and *j*th nodes, i≠j), the mean $m_{ij}^{k}$, covariance $C_{ij}^{k}$, and weight $w_{ij}^{k}$ are generated as described in [5,6]:

(5) $$\begin{array}{cc} m_{ij}^{k} & =\left[\begin{array}{c} x_{i}\left(n\right)+d_{ij}\cos\left(\frac{2\pi k}{N}\right)\\ y_{i}\left(n\right)+d_{ij}\sin\left(\frac{2\pi k}{N}\right) \end{array}\right], \end{array}$$

(6) $$\begin{array}{cc} C_{ij}^{k} & =\left[\begin{array}{cc} v_{r} & v_{t} \end{array}\right]\left[\begin{array}{cc} \sigma_{r}^{2} & 0\\ 0 & \sigma_{t}^{2} \end{array}\right]\left[\begin{array}{c} v_{r}^{T}\\ v_{t}^{T} \end{array}\right], \end{array}$$

(7) $$\begin{array}{cc} w_{ij}^{k} & =\frac{1}{N}, \end{array}$$

where k=0,1,…,N−1, is an index of SoG, and *N* is number of SoG, respectively. Note that the procedures of generating the SoG variables (i.e., Equations (5)–()) is utilized in Algorithms 1 and 2.

#### 2.2.3. Updating SoG

The update scheme of SoG variables consists of two parts. When collecting the current measurement $d_{ij}$, the corresponding mean $m_{ij}^{k}$ and covariance $C_{ij}^{k}$ are first updated using the standard EKF procedure (see [15]). After that, the weights $w_{ij}^{k}$ between all inter-nodes are updated by using the inter-node measurements to remove the ambiguity.

A detailed update procedure for the weight $w_{ij}^{k}$ is summarized in Algorithm 3. In order to update $w_{ij}^{k}$ between the *i*th and *j*th nodes, the likelihood $l_{ij,m}^{k}$ is first computed with respect to the *m*th nodes connected with the *j*th node (in lines 2–5 of Algorithm 3), where *m* is an index of the neighbor nodes. After the normalization of $l_{ij,m}^{k}$ in lines 6–8, the weight $w_{ij}^{k}$ is updated in lines 9–11 and normalized in lines 12–14. The above procedures are performed for all possible *m*th neighbor nodes, i.e., m≠i, m≠j. As more measurements $d_{mj}$ are used, the updated weight $w_{ij}^{k}$ becomes a larger value if $d_{ij,m}^{k}$ is close to $d_{mj}$; otherwise, it will converge rapidly to zero.
**Algorithm 3:** Weight update procedure.
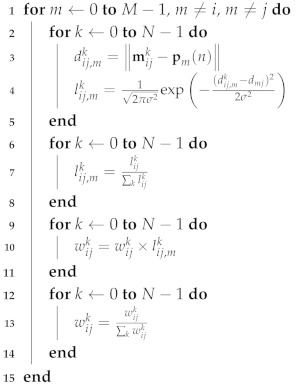


#### 2.2.4. Merging SoG

After updating the SoG variables, the weighted SoG is merged into a single Gaussian distribution to estimate the new locations of nodes. A closed-form solution [17,18] for the merged SoG is employed for the new location $p_{i}\left(n+1\right)$ and its covariance $C_{i}\left(n+1\right)$, as follows:

(8) $$\begin{array}{cc} p_{i}\left(n+1\right) & =\frac{1}{\sum_{j,k}\left(w_{ji}^{k}\right)}\sum_{j,k}\left(w_{ji}^{k}m_{ji}^{k}\right), \end{array}$$

(9) $$\begin{array}{cc} C_{i}\left(n+1\right) & =\frac{1}{\sum_{j,k}\left(w_{ji}^{k}\right)}\sum_{j,k}w_{ji}^{k}\left(C_{ji}^{k}+{\bar{C}}_{ji}^{k}\right), \end{array}$$

(10) $$\begin{array}{cc} {\bar{C}}_{ji}^{k} & =\left(m_{ji}^{k}-p_{i}\left(n+1\right)\right){\left(m_{ji}^{k}-p_{i}\left(n+1\right)\right)}^{T}. \end{array}$$

#### 2.2.5. Transferring Weight

Due to the inherent non-convex nature of anchor-free localization, a local solution can be obtained after merging SoG variables. To prevent falling into a local solution due to a small weight being ignored, a weight transfer method is introduced here by distributing weights around the maximum weight. If $k_{\max}$ indicates an index of the maximum weight from the SoG distribution, the weight can be heuristically distributed as a Gaussian distribution at the center of $k_{\max}$. More specifically, with the mean distance between two indices $k_{\max}$ and *k*, defined by $d_{k_{\max}-k}=∥m_{ij}^{k_{\max}}-m_{ij}^{k}∥$, the weight is distributed as follows:

(11) $$w_{ij}^{k}=\frac{1}{\sqrt{2\pi \sigma^{2}}}\exp\left(-\frac{d_{k_{\max}-k}^{2}}{2\sigma^{2}}\right),$$

where σ is experimentally determined (e.g., 0.5). Finally, the weight $w_{ij}^{k}$ is normalized to ensure that its sum is 1.

**Figure 5:**
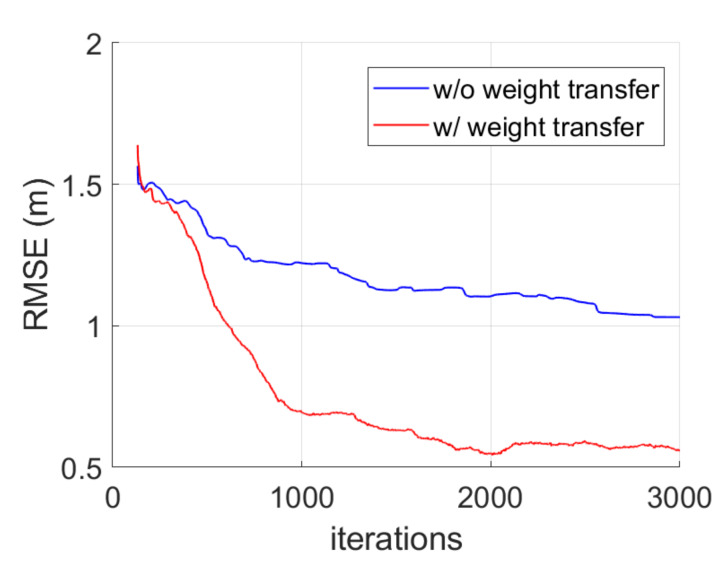
The root-mean-square error (RMSE) performance of map estimation with and without the weight transfer technique.

The effectiveness of the proposed weight transfer technique was verified with a simulation, as shown in Figure 5. In the simulation, five nodes were deployed, where four nodes were placed in a rectangular shape and a robot was moved inside the rectangular shape. The noise variance was set to 0.3--0.8. The RMSE performance was obtained by ensemble averaging over 20 independent trials. As shown in Figure 5, the RMSE performance improved by about 50% with the proposed weight transfer method because it gives a chance to avoid a local minimum.

#### 2.2.6. Convergence

The convergence of each node is determined by using ${\tilde{p}}_{i}\left(n\right)$, the time-average of $p_{i}\left(n\right)$, as follows:

(12) $${\tilde{p}}_{i}\left(n+1\right)=\alpha {\tilde{p}}_{i}\left(n\right)+\left(1-\alpha \right)p_{i}\left(n\right).$$

If $∥{\tilde{p}}_{i}\left(n+1\right)-{\tilde{p}}_{i}\left(n\right)∥<\varepsilon$, then the corresponding node is considered to be converged. Here, ε is a small positive constant threshold. If not converged, the procedure from Section 2.2.3 to Section 2.2.4 is repeated when a new measurement is available.

### 2.3. Moving Stage

As mentioned in Section 2.1, the movement of any node can be tracked in the moving stage. Unlike the conventional RO-SLAM algorithms, the proposed CDRO-SLAM can deal with the movement of the neighbor nodes as well as the robot’s movement. In particular, the tracking procedure during the movement of neighbor nodes was derived in Algorithm 2 by adopting a detection and tracking moving object (DATMO) strategy [19] into a SoG approach. As shown in Algorithm 2, the same procedures as in the initialization stage are recombined; thus, the detailed procedure for the moving stage is not given here. Before concluding this section, it should be noted from Algorithm 2 that the indices of SoG variables in (ii) and (iii) of Algorithm 2 are symmetric counterparts of those in (v) and (vi) (see Figure 2b,c), which will be utilized when developing an efficient implementation, as described in the following section.

## 3. Efficient Implementation

Due to the extra SoG variables between the inter-nodes, the CDRO-SLAM [14] has a greater computational cost compared with the conventional SoG based RO-SLAM algorithms which use only the direct measurements between a robot and its neighbor nodes. To mitigate this incurred computational burden, several efficient implementation techniques are proposed here.

Efficient implementation can be applied to the corresponding computation parts shown in Algorithms 1–3. Especially, the weight update procedure that causes the main computational burden is summarized in Algorithm 3 for the original one and Algorithm 4 for the efficient implementation to show the computational efficiency.
**Algorithm 4:** Efficient weight update procedure.
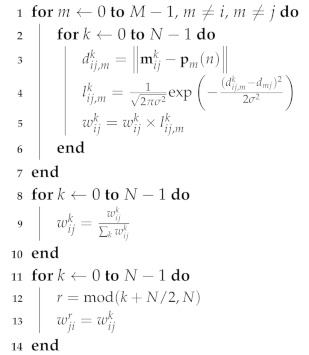


### 3.1. Reduced Normalization

When computing the likelihood $l_{ij,m}^{k}$, its normalization can be omitted without damaging the performance. Similarly, the normalization of the weight $w_{ij}^{k}$ can be carried out only once after the end of for-loop *m*.

### 3.2. Symmetric SoG Distribution

**Figure 6:**
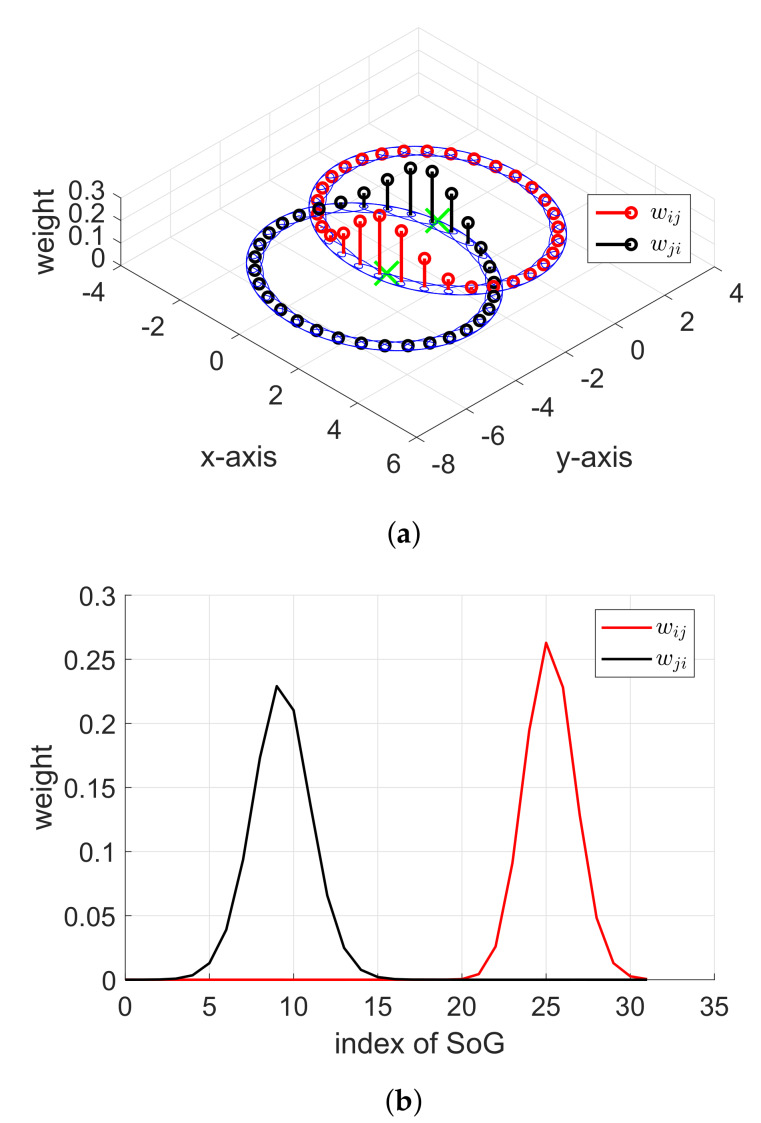
Weight symmetry between $w_{ij}^{k}$ and $w_{ji}^{k}$. The green crosses represent the estimated positions of the *i*th and *j*th nodes. (**a**) The SoG variables are distributed in the xy plane, and the weight is shown on the z-axis. (**b**) The weights $w_{ij}^{k}$ and $w_{ji}^{k}$ are depicted against the SoG index k.

Obviously, a higher weight represents a higher probability of the existence of a node. Let us consider two weights $w_{ij}^{k}$ and $w_{ji}^{k}$ for i≠j. As shown in Figure 6a, the high weight area for each node is symmetrical, i.e., a high $w_{ij}^{k}$ occurs in the direction of a negative y-axis for the $w_{ij}^{k}$ distribution, and a high $w_{ji}^{k}$ occurs in the direction of the positive y-axis for the $w_{ji}^{k}$ distribution.

In Figure 6b, the calculated distribution of two weights $w_{ij}^{k}$ and $w_{ji}^{k}$ is described when N=32 without considering symmetry. If the symmetry property is used, once $w_{ij}^{k}$ is computed (the highest probability occurred in k=25), its counterpart $w_{ji}^{k}$ can be simply copied from $w_{ij}^{k}$ by using the modulo operation (shown in lines 11–14 of Algorithm 4), which gives k=9 for the highest probability and is equal to the index with the calculated distribution of $w_{ji}^{k}$ in Figure 6b.

Furthermore, the symmetry property can be applied in the movement stage. As discussed in the previous section, the SoG distributions of (iii) and (vi) in Algorithm 2 are symmetrical counterparts of each other. Therefore, the weight $w_{ji}^{k}$ of (vi) of Algorithm 2 is simply symmetrically copied from the weight $w_{ij}^{k}$ that was previously computed in (iii) of Algorithm 2.

### 3.3. Removal of Low Probability

As investigated in several SoG based RO-SLAM algorithms, the merging procedure can be efficiently realized by dropping small Gaussian weights in Equations (8)–(). The threshold value can be set as small enough, e.g., 0.00001/N [20], where *N* is the number of SoG.

### 3.4. Efficiency Analysis

In order to show the efficiency, two versions of the proposed algorithm are considered: one is the original CDRO-SLAM and the other is its efficient implementation version (eCDRO-SLAM) with the aforementioned efficient techniques. The computational efficiency of CDRO-SLAM and eCDRO-SLAM is also compared with that of several conventional RO-SLAM algorithms, i.e., the efficient probabilistic RO-SLAM (EPRO-SLAM) [6] and the RBPF-based RO-SLAM (RBPF-RO-SLAM) algorithm [7].

The required computational complexity for updating the weight is considered in terms of the computing likelihood number $l_{ij,m}^{k}$, which is cost-dominant and is summarized in Table 1. Here, *M*, *Q*, and *N* are the numbers of nodes, particles, and SoG, respectively. Note that RBPF-RO-SLAM requires a higher computational cost than EPRO-SLAM, since $Q=\beta \cdot d_{ij}$ is usually determined to be much larger than *N*, e.g., Q≥1000 and N=32, where β=[400,2000] [7].

For CDRO-SLAM, Algorithm 3 requires N(M−2) computations, and for eCDRO-SLAM, Algorithm 4 needs N(M−2)/2 computations, on average. The computation of Algorithm 4 is reduced by half compared with Algorithm 3.

It is not easy to compare non-cooperative and cooperative algorithms at the same level because their approaches to estimating the location are totally different, i.e., the cooperative algorithms use many inter-node measurements for the map estimation, but the non-cooperative algorithms use robot movement to measure independent and direct ranges between the robot and its neighbors. As illustrated in Figure 1a–c, the EPRO-SLAM requires several direct measurements at the non-collinear locations to remove the ambiguity in the location of nodes, which results in αN computations in total, where α is the number of required direct measurements at the non-collinear locations. On the other hand, the proposed CDRO-SLAM can yield an accurate location even with one iteration, as described in Figure 3a–c. In this case, the computational burden ratio of the proposed eCDRO-SLAM over EPRO-SLAM is $\frac{(M-2)/2}{\alpha }$ at each iteration. When α is minimal (i.e., α=3 as shown in Figure 1), the computational complexity ratio can be approximately $\frac{M}{6}$. In the case of (at least) α≈M, to achieve a similar ambiguity level to the proposed eCDRO-SLAM, the ratio can be approximately $\frac{1}{2}$.

Furthermore, when updating all nodes, the total computational burden of the proposed eCDRO-SLAM is NM(M−1)(M−2)/2, since there are M(M−1) SoG variables between inter-nodes; thus, the total computational burden ratio is about $\frac{NM(M-1)(M-2)/2}{NM\alpha }\approx M/2$ for α≈M, i.e., the total computational burden increases linearly as the number of nodes, *M*, increases.

## 4. Experiments

**Figure 7:**
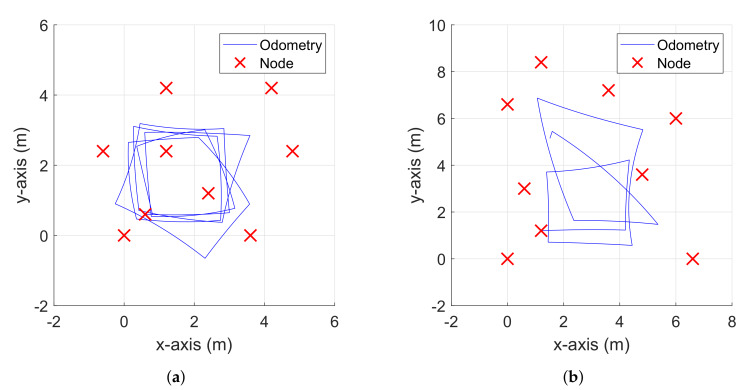
Deployment of nodes and odometry for (**a**) Experiment 1 and (**b**) Experiment 2.

As shown in Figure 7, two sets of experiments were conducted with the same number of nodes (M=9) but different sizes (3.6 m × 4.8 m for Experiment 1 and 6.6 m × 8.4 m for Experiment 2). In the experiments, the motion of the robot was arbitrary controlled remotely by a person, and it was represented by using the measured odometry in Figure 7. Also, TurtleBot3 Burger and Pozyx [21] were used in the experiments for the ultra-wide-band (UWB) measurements. For the acquisition of measurements, all the inter-node measurements were initially collected while a robot remained still, and then the direct measurements were collected while the robot moved, in order to clearly show the difference of weight update scheme between the conventional RO-SLAM and the proposed eCDRO-SLAM. The performance of the eCDRO-SLAM with the inter-node measurements was verified with real experimental data, and it was compared with EPRO-SLAM [6] and RBPF RO-SLAM [7], which use direct measurements only. Note that the proposed eCDRO-SLAM was considered only because it gave almost same performance when compared with the CDRO-SLAM, except the computational efficiency. The computational complexity comparison of the eCDRO-SLAM and CDRO-SLAM will be given in Section 4.3. The map estimation and the tracking performance of moving nodes were also investigated.

### 4.1. Map Estimation Performance

**Figure 8:**
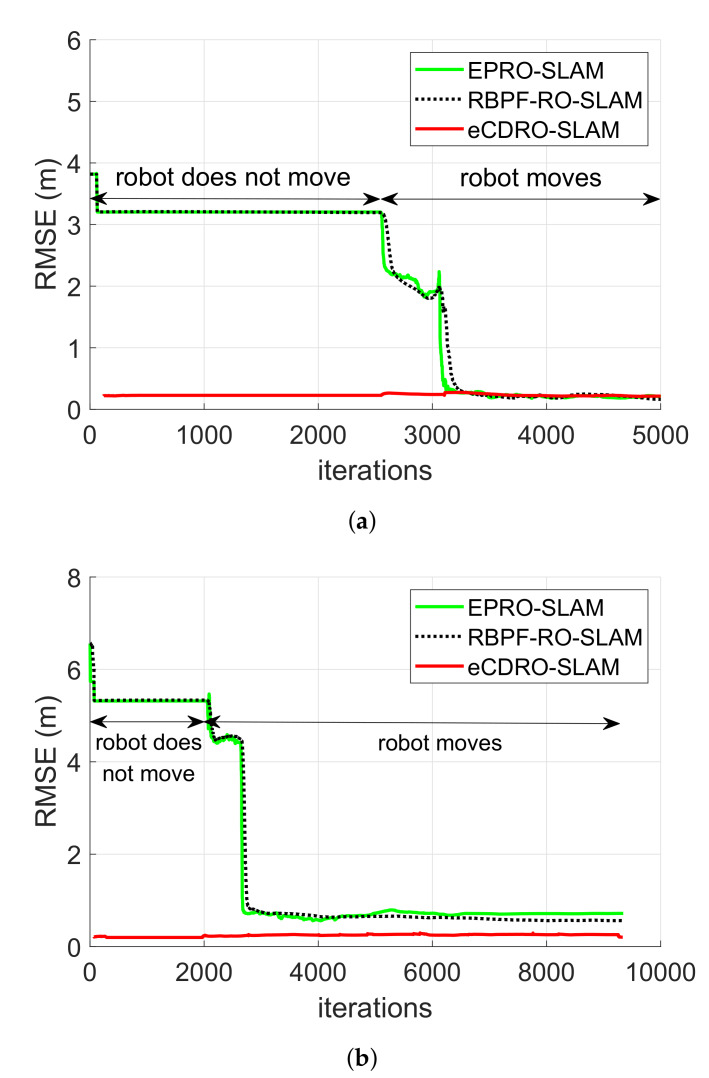
Map estimation performance obtained by the proposed eCDRO-SLAM and the conventional RO-SLAM algorithms [6,7] for (**a**) Experiment 1 and (**b**) Experiment 2.

The map estimation performance for the two experiments is illustrated in Figure 8, and the following results can be observed:

- The conventional algorithms exhibited a slower convergence rate and higher initial map estimation error than the proposed eCDRO-SLAM, since their convergence depends on both the movement of the robot and the rate of measurements. On the other hand, the convergence of the proposed eCDRO-SLAM depends only on the rate of measurements and is accelerated by employing the inter-node measurements, as discussed before.
- As shown in Figure 8, the proposed eCDRO-SLAM estimates the map even without the movement of a robot; on the other hand, the conventional algorithms can only estimate the map when a robot moves.
- During the refinement of Experiment 1, all algorithms achieved a similar RMSE of about 0.2 m. On the other hand, for Experiment 2, the RBPF-RO-SLAM algorithm (0.56 m) yielded a higher RMSE than the EPRO-SLAM algorithm (0.72 m), as expected. Furthermore, the proposed eCDRO-SLAM algorithm (0.21 m) obtained a better performance than the non-cooperative RBPF-RO-SLAM algorithm. The difference between the two experiments comes from the odometry error. The performance of the RBPF-RO-SLAM (also the EPRO-SLAM) suffered from not only the measurement error but also the odometry error, especially in the large area; on the other hand, the proposed eCDRO-SLAM was affected only by the measurement error, which led to a similar map estimation result for both experiments, regardless of the difference in size.

### 4.2. Node Tracking Performance

**Figure 9:**
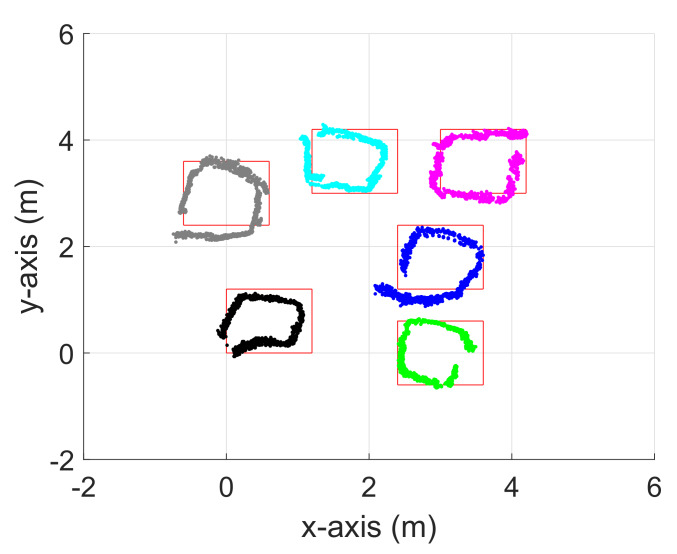
Node tracking performance of the eCDRO-SLAM for Experiment 1.

The node tracking performance of the proposed eCDRO-SLAM was verified with Experiment 1. Each node moves continually in a square shape one-by-one. The tracking performance was measured after the initialization stage, and its result is illustrated in Figure 9. The average localization error was about 0.3688 m in the movement stage, somewhat larger than that of the initial stage; however, a reasonable tracking performance was achieved using the range sensor only. The tracking performance of the proposed eCDRO-SLAM can be further improved if a node moves in a stop-and-go way or if some sensors such as the inertial measurement unit (IMU) are fused. On the other hand, the most conventional RO-SLAM algorithms (including EPRO-SLAM [6] and RBPF-RO-SLAM [7]) cannot be applied to dynamic environments where nodes can have motion.

### 4.3. Computational Complexity

In Table 2, the computational complexity of the proposed algorithm and the conventional algorithms is summarized in terms of the average CPU time per sample for updating the weight $w_{ij}^{k}$ and for executing the complete algorithm. The CPU time was measured through implementation in a MATLAB simulation with an Intel Core i7 processor of 3.3 GHz and 8 GB memory.

The required CPU time for computing the weight shown in Table 2 has a similar tendency to that of the efficiency analysis shown in Section 3.4. When considering the total CPU time, it is assumed that all algorithms spend most of the CPU time in updating the weight and merging SoG variables (or particles) to simplify the analysis. From the total CPU time results, we can see the following:

- The total computational burden ratio of the proposed eCDRO-SLAM over the CDRO-SLAM (9.20/22.7≈0.405) is smaller than the expected ratio of the weight (0.101/0.170≈0.594), since the eCDRO-SLAM is efficiently implemented for both updating and merging SoG processes. As indicated in Section 3.4, the efficiency of the eCDRO-SLAM is about half that of the CDRO-SLAM.
- The eCDRO-SLAM requires 9.20−0.101×(9×8)=1.93 ms to merge SoG variables between all inter-nodes, and the EPRO-SLAM needs 1.70−0.032=1.67 ms to merge only one SoG distribution. This efficiency of the eCDRO-SLAM is obtained due to the fast convergence of the weight and because small enough weights are dropped in the merging process. Furthermore, the eCDRO-SLAM is computationally efficient in terms of achieving a similar ambiguity level, since the EPRO-SLAM may require M=9 iterations (see Section 3.4) and, in this case, it needs 1.7 ms × 9 = 15.3 ms in total.
- Despite the cooperative approach, the proposed eCDRO-SLAM was executed using a smaller total CPU time than the non-cooperative RBPF-RO-SLAM. Note that the RBPF-RO-SLAM spends most of the CPU time for transforming particles into a Gaussian filter.

## 5. Conclusions

In this paper, an efficient cooperative dynamic RO-SLAM (eCDRO-SLAM) based on the sum of Gaussian (SoG) filter integrating all inter-node measurements for the localization was proposed. A new framework to update the SoG variables using the inter-node measurements leads to faster map estimation with less error than conventional RO-SLAM algorithms using the direct measurements only. Also, several efficient implementation techniques such as the weight symmetry of the SoG distributions were introduced to alleviate the additional computational burden induced when calculating the SoG variables between inter-nodes.

Furthermore, the proposed eCDRO-SLAM was designed to track the movement of some nodes by resetting and updating the SoG variables. With the dynamic property and computational efficiency, its application can be extended to various dynamic environments that are full of movable objects such as unstable disaster sites, unstructured fields, or temporal deployment of robots/devices.

As a future work, a sensor fusion approach integrating the IMU, odometry, and range sensor will be considered to improve the localization and node tracking performance. Furthermore, we will apply the proposed algorithm to various RO-SLAM applications such as robot-assisted search and rescue application and ambient intelligence environments.

## Figures and Tables

**Table 1:** Computational complexity of updating the weight.

Algorithms	Likelihood
EPRO-SLAM [6]	*N*
RBPF-RO-SLAM [7]	*Q*
CDRO-SLAM	N(M−2)
eCDRO-SLAM	N(M−2)/2

**Table 2:** Required CPU time for Experiment 1.

Algorithms	CPU Time (ms)	
	Weight	Total
EPRO-SLAM [6]	0.032	1.70
RBPF-RO-SLAM [7]	0.415	13.3
CDRO-SLAM	0.170	22.7
eCDRO-SLAM	0.101	9.20
